# Comparing Facebook Users and Facebook Non-Users: Relationship between Personality Traits and Mental Health Variables – An Exploratory Study

**DOI:** 10.1371/journal.pone.0166999

**Published:** 2016-12-01

**Authors:** Julia Brailovskaia, Jürgen Margraf

**Affiliations:** Mental Health Research and Treatment Center, Ruhr-Universität Bochum, Bochum, Germany; University of Granada, SPAIN

## Abstract

Over one billion people use Facebook as a platform for social interaction and self-presentation making it one of the most popular online sites. The aim of the present study was to investigate differences in various personality traits and mental health variables between Facebook users and people who do not use this platform. The data of 945 participants (790 Facebook users, 155 Facebook non-users) were collected. Results indicate that Facebook users score significantly higher on narcissism, self-esteem and extraversion than Facebook non-users. Furthermore, they have significantly higher values of social support, life satisfaction and subjective happiness. Facebook non-users have (marginally) significantly higher values of depression symptoms than Facebook users. In both groups, extraversion, self-esteem, happiness, life satisfaction, resilience and social support, on the one hand, and depression, anxiety and stress symptoms, on the other hand, are negatively correlated. Neuroticism is positively associated with depression, anxiety and stress symptoms. However, significant differences exist between Facebook users and Facebook non-users regarding some associations of personality traits and mental health variables. Compared to Facebook non-users, the present results indicate that Facebook users have higher values of certain personality traits and positive variables protecting mental health. These findings are of particular interest considering the high importance of social online-platforms in the daily life of many people.

## Introduction

Today, over 1.5 billion people are members of the social networking site (SNS) Facebook [1]. Facebook is a multifunctional online community, where users can lead an online life similar to their life offline or completely different from it [2,3]. On average, users spend fifty minutes daily on Facebook [4] presenting themselves through different actions, for example uploading photos and writing status updates [5–10].

Recent studies on Facebook use showed that certain personality traits are related to online behavior [11]. Extraverted people spend a lot of time on Facebook, upload many photos and have extraordinarily long lists of online-friends [12,13]. The studies found a negative relationship between users’ level of agreeableness and the time spent on Facebook as well as the number of online-friends and the number of uploaded photos [14].

Studies investigating the relationship between self-esteem and online behavior showed contradictory results. In a study conducted on Facebook use, users with low self-esteem spent more time daily on Facebook and had more attractive photos than those with a high level of self-esteem [15]. However, the level of self-esteem was positively related to the number of online-friends and discussion groups in users of the German social network StudiVZ [16]. Similarly in those using the Chinese network Renren, the level of self-esteem was positively related to the frequency of commenting on status updates [17].

Persons with a high level of narcissism have a positive and inflated self-view, self-love, self-serving bias, sense of entitlement, self-importance and uniqueness [18–21]. On SNSs such as Facebook, these individuals have many online-friends, frequently write status-updates, join many discussion groups and choose attractive profile photos for their pages [15,22–25].

Regarding the considerable amount of time spent on Facebook, it seems likely that not only personality traits of users are related to online behavior, but so is also their mental health.

Studies investigating the relationship between Facebook use and mental health variables have described a positive relationship between Internet use, e.g., chatting or e-mailing, and mental disorders [26]. Most of these studies found depression to be positively associated with online behavior, especially the use of SNSs like Facebook [27–31]. A positive correlation between depression and time spent on Facebook has been described [32]. In contrast, some studies found no relationship between depression and SNSs use [33]. Furthermore, a positive relationship was found between the Narcissistic Personality Disorder and Facebook use, e.g., number of online-friends [34].

Regardless of the dual-factor model of mental health [35,36] which describes positive mental health and mental illness (negative mental health) as two interrelated but separate unipolar dimensions of mental health [37], most studies that investigated the association between mental health and the use of social networks focused only on mental disorders. However, the relationship between the use of SNSs and positive mental health variables, such as happiness, resilience, life satisfaction and social support [38–41] is seldom investigated, and the available results are inconsistent. While some studies have shown a positive relationship between well-being, happiness and SNSs use [42–46], other studies described a negative association between these variables [47].

Furthermore, most authors investigated only SNSs users, for example users of Facebook, in their studies on the relationship between mental health variables or personality traits and online behavior. Comparisons between individuals who are users of a specific SNS and those who do not use the particular SNS are rare. Studies examining such comparisons showed significant differences. While Facebook users had higher values of narcissism and extraversion, Facebook non-users showed higher values of conscientiousness [48,49].

Considering the described results of earlier studies and the high impact of SNSs like Facebook on people’s everyday life, the present study aimed to fill this gap in research by comparing Facebook users and Facebook non-users regarding the personality traits narcissism, self-esteem and the “Big Five” (neuroticism, extraversion, openness to experiences, agreeableness, conscientiousness) and mental health variables. Considering the dual-factor model of mental health [35–37], we aimed to investigate negative (depression symptoms, anxiety symptoms, stress symptoms) as well as positive (subjective happiness, resilience, life satisfaction, social support) variables of mental health.

Given that only little is known about differences in mental health (negative factors, such as anxiety; positive factors, such as life satisfaction, see also [50]) between Facebook users and Facebook non-users, the natures of the current study is exploratory. Based on the knowledge about the investigated constructs, we expected Facebook users to have higher values of narcissism (Hypothesis 1a) and extraversion (Hypothesis 1b) than Facebook non-users. The level of conscientiousness was assumed to be higher in the group of Facebook non-users (Hypothesis 1c). Depression was expected to be higher in the group of Facebook users (Hypothesis 2). Resilience, social support, happiness and life satisfaction were assumed to be associated with depression, anxiety and stress in both groups (Hypothesis 3).

Furthermore, we investigated whether the levels of self-esteem, neuroticism, agreeableness, anxiety and stress, as well as happiness, resilience, life satisfaction and social support differ between Facebook users and Facebook non-users. Also, the relationship between narcissism, self-esteem and the “Big Five”, on the one hand, and depression, anxiety and stress symptoms, on the other hand, was analyzed.

## Materials and Methods

### Procedure and participants

The present study belongs to the ongoing BOOM (Bochum Optimism and Mental Health) project aiming to investigate risk and protective factors of mental health [50–52]. All data of the present study were collected with an online self-report questionnaire on the research platform www.unipark.de. We sent a collective e-mail invitation to all students of the Ruhr-Universität Bochum containing a link to the online questionnaire. Participation was possible between October 2015 and December 2015. Research and Ethics Committee approval of the Ethics Committee of the Faculty of Psychology of the Ruhr-Universität Bochum for the implementation of the study was received. We followed all national regulations and laws regarding human subjects research, and obtained the required permissions to conduct the present study. Participants were properly instructed and gave online informed consent to participate. The dataset used in the present study is available in S1 Dataset.

The total sample included 945 students of the Ruhr-Universität Bochum. 790 participants (228 men, 562 women; age (years): *M* = 23.42, *SD* = 5.02, range: 17–58) were identified as Facebook users. 155 participants (56 men, 99 women; age (years): *M* = 25.28, *SD* = 6.35, range: 17–52) were not members of the SNS Facebook.

### Measures

To assess happiness, the Subjective Happiness Scale (SHS) was used [53]. The one-dimensional questionnaire consisted of four items rated on a 7-point Likert scale (range: 1–7; scale reliability: Cronbach’s alpha = .82).

The Satisfaction with Life Scale (SWLS) was used to measure global life satisfaction [54]. The five items of this one-dimensional questionnaire were rated on a 7-point Likert scale (1 = strongly disagree; 7 = strongly agree; scale reliability: Cronbach’s alpha = .92) [55].

To measure resilience, the one-dimensional German Resilience Scale 11 (RS-11) was used [56,57]. Participants rated 11 items on a 7-point Likert scale (1 = disagree; 7 = agree; scale reliability: Cronbach’s alpha = .91).

Subjective experienced or anticipated support from the social network was measured with the one-dimensional German Questionnaire Social Support (F-SozU K-14) [58] consisting of 14 items rated on a 5-point Likert scale (1 = not true; 5 = true; scale reliability: Cronbach’s alpha = .94).

Narcissism was measured with the 13 force-choice format items of the Narcissistic Personality Inventory 13 (NPI-13; scale reliability: Cronbach’s alpha = .82) [59].

The personality traits of the “Big Five” were assessed with the Big Five Inventory 10 (BFI-10) [60]. The ten items were rated on a 5-point Likert scale (1 = disagree strongly; 5 = agree strongly). Respectively, two items belonged to one of the five scales extraversion (scale reliability: Cronbach’s alpha = .89), agreeableness (Cronbach’s alpha = .74), conscientiousness (Cronbach’s alpha = .82), neuroticism (Cronbach’s alpha = .86), and openness (Cronbach’s alpha = .79).

To measure the participants’ self-esteem, the German version of the Single-Item Self-Esteem Scale (SISE) was used [61]. Participants were asked to rate on a 5-point Likert scale how much the statement “I have high self-esteem.” applied to themselves (1 = not very true of me; 5 = very true of me).

The Depression Anxiety Stress Scales 21 (DASS-21) were applied to measure depression, anxiety and stress symptoms [62,63]. The whole questionnaire consisted of three reliable 7-item scales, which were rated on 4-point Likert scales (0 = did not apply to me at all; 3 = applied to me very much or most of the time; scale reliability: depression: Cronbach’s alpha = .83, anxiety: Cronbach’s alpha = .78, stress: Cronbach’s alpha = .87) [64,65].

Furthermore, participants were asked about their social media use. First, Internet use was measured (“How often do you use the Internet as a whole?”). In the next step, the SNSs use was assessed (“How often do you use social networking sites?”). The answer for both questions was given on a 7-point Likert scale (0 = never; 1 = less than once a month; 2 = once or twice a month; 3 = once a week; 4 = once or twice a week; 5 = once a day; 6 = more than once a day). Then, participants were asked whether they are a member of the SNSs Facebook, Twitter, Instagram, Tumblr, Google+ or any other platform (0 = no; 1 = yes).

### Statistical analyses

The Statistical Package for the Social Sciences (SPSS) version 21 was used for all statistical analyses [66,67]. First, the means of the investigated personality traits and mental health variables were compared between Facebook users and Facebook non-users by calculating multivariate analysis of variance (MANOVA). Following Steven’s [67] recommendation not to use more than ten dependent variables in one MANOVA, we conducted two MANOVAs. The first analysis included self-esteem, narcissism and the “Big Five” traits. Since Box’s test was non-significant, we used Pillai’s trace as a multivariate test. The second analysis included the mental health variables life satisfaction, resilience, social support, happiness, depression, anxiety, and stress. Here, Box’s test was significant, therefore, the Hotelling’s trace statistics were used. However, all four multivariate tests showed similar results.

Next, the association between the investigated variables was assessed by zero-order bivariate correlations separately in each of the groups. Further, we conducted two multiple linear regression analyses. First, the variables age, gender, self-esteem, narcissism and the traits of the “Big Five” were taken as independent variables, and depression, anxiety and stress, successively, as dependent variables. Next, the variables age, gender, happiness, life satisfaction, resilience and social support served as independent variables, and depression, anxiety and stress, successively, as dependent variables.

## Results

All investigated variables were close to normally distributed (indicated by Kolmogorov-Smirnov test, analyses of skew, kurtosis, and histogram).

Table 1 shows the descriptive statistics of the social media use of both groups. All participants used the Internet. 93.4% of the Facebook users and 89% of the Facebook non-users used the Internet daily (sum of “more than once a day” and “once a day”). SNSs in general were used by 54.8% of the Facebook users daily. 53.5% of the Facebook non-users used SNSs in varying frequency, e.g., 5.8% used them daily and 22.6% less than once a month. Facebook users reported memberships on up to four other SNSs, in particular Instagram. Facebook non-users were members on up to three different SNSs. Most of them were members on Twitter, followed by Instagram and Tumblr.

**Table 1:** Descriptive statistics of social media use of Facebook users and Facebook non-users.

	Facebook users (N = 790)	Facebook non-users (N = 155)
	N (%)	N (%)
**Internet use**		
(0) “never”	0	0
(1) “less than once a month”	3 (0.4)	1 (0.6)
(2) “once or twice a month”	2 (0.3)	2 (1.3)
(3) “once a week”	15 (1.9)	2 (1.3)
(4) “once or twice a week”	32 (4.1)	12 (7.7)
(5) “once a day”	101 (12.8)	22 (14.2)
(6) “more than once a day”	637 (80.6)	116 (74.8)
**SNSs use**		
(0) “never”	0	72 (46.5)
(1) “less than once a month”	51 (6.5)	35 (22.6)
(2) “once or twice a month”	43 (5.4)	15 (9.7)
(3) “once a week”	92 (11.6)	12 (7.7)
(4) “once or twice a week”	171 (21.6)	12 (7.7)
(5) “once a day”	155 (19.6)	4 (2.6)
(6) “more than once a day”	278 (35.2)	5 (3.2)
**SNSs membership**		
Facebook	790 (100.0)	0
Twitter	79 (10.0)	19 (12.3)
Instagram	205 (25.9)	17 (11.0)
Tumblr	52 (6.6)	17 (11.0)
Google+	45 (5.7)	6 (3.9)
Any other platform	46 (5.8)	12 (7.7)

SNSs = social networking sites; due to rounding, the sum of listed figures may be higher/lower than 100%.

### Comparison of personality traits and mental health variables between Facebook users and Facebook non-users

Table 2 presents the descriptive statistics of the measured constructs separately for Facebook users and Facebook non-users as well as the results of the two MANOVAs. First, self-esteem, narcissism and the “Big Five” traits were compared between the two groups. Pillai’s trace was significant, V = .037, F(7,937) = 5.101, p < .001, partial eta squared = .037, demonstrating significant differences between the two groups. Then, both groups were compared regarding the mental health variables. Hotelling’s trace was significant, T = .028, F(7,937) = 3.792, p < .001, partial eta squared = .028. Thus, also the level of the mental health variables differed between the groups.

**Table 2:** Means, standard deviations, mean comparisons of personality traits and of mental health variables between Facebook users and Facebook non-users.

	Facebook users (N = 790)	Facebook non-users (N = 155)		
	M (SD)	M (SD)	F	p
**1. MANOVA**				
SISE	3.39 (1.04)	3.06 (1.23)	11.695	< .01
NPI-13	3.93 (2.29)	3.44 (2.21)	5.953	< .05
BFI-10: Extraversion	6.43 (1.96)	5.65 (2.18)	19.558	< .001
BFI-10: Agreeableness	6.44 (1.59)	6.30 (1.73)	.976	.324
BFI-10: Conscientiousness	6.95 (1.75)	7.05 (1.86)	.443	.506
BFI-10: Neuroticism	6.29 (1.90)	6.24 (2.09)	.070	.792
BFI-10: Openness	7.39 (1.95)	7.60 (2.01)	1.541	.215
**2. MANOVA**				
SWLS	25.03 (6.19)	23.61 (6.78)	6.598	< .05
RS-11	59.69 (8.91)	58.17 (10.86)	3.528	.061
F-sozU K-14	60.75 (9.22)	57.89 (11.41)	11.428	< .01
SHS	18.79 (5.10)	17.39 (5.93)	9.180	< .01
DASS-21: Depression	4.36 (4.50)	5.14 (5.22)	3.727	.054
DASS-21: Anxiety	3.39 (3.80)	3.70 (4.46)	.776	.379
DASS-21: Stress	6.41 (4.76)	5.98 (5.08)	1.020	.313

SISE = Singe-Item Self-Esteem Scale; NPI-13 = Narcissistic Personality Inventory 13; BFI-10 = Big Five Inventory 10; SWLS = Satisfaction with Life Scale; RS-11 = Resilience Scale 11; F-sozU K-14 = Questionnaire Social Support; SHS = Subjective Happiness Scale; DASS-21 = Depression Anxiety Stress Scales 21; MANOVA = multivariate analysis of variance; M = mean; SD = standard deviation; degrees of freedom of all F-values = 1,943; p = significance.

Results reveal that Facebook users had significantly higher means of self-esteem, narcissism, extraversion, life satisfaction, social support and subjective happiness than Facebook non-users. In contrast, Facebook non-users showed marginally higher values of depression symptoms. We found no significant differences for the means of resilience, agreeableness, conscientiousness, neuroticism, openness, anxiety and stress symptoms.

### Correlations: Personality traits and mental health variables

In both groups, extraversion and self-esteem were significantly negatively related to depression, anxiety and stress symptoms. Conscientiousness correlated significantly negatively with depression. In contrast, neuroticism correlated significantly positively with depression, anxiety and stress symptoms.

Only in the Facebook user group, agreeableness was significantly negatively related to depression and stress symptoms (see Table 3).

**Table 3:** Correlations of personality traits, life satisfaction, resilience, social support and happiness with depression, anxiety and stress symptoms (Facebook users, Facebook non-users).

	Facebook users (N = 790)			Facebook non-users (N = 155)		
	Depression	Anxiety	Stress	Depression	Anxiety	Stress
SISE	-.515**	-.413**	-.409**	-.501**	-.425**	-.420**
NPI-13	-.061	-.052	.031	-.016	-.047	.020
BFI-10: Extraversion	-.293**	-.232**	-.162**	-.378**	-.277**	-.241**
BFI-10: Agreeableness	-.141**	-.039	-.154**	-.088	.044	-.007
BFI-10: Conscientiousness	-.169**	-.058	-.009	-.182	-.078	-.027
BFI-10: Neuroticism	.341**	.418**	.508**	.409**	.409**	.512**
BFI-10: Openness	-.024	-.053	-.023	-.082	-.026	-.049
SWLS	-.564**	-.374**	-.416**	-.609**	-.468**	-.495**
RS-11	-.466**	-.339**	-.333**	-.589**	-.503**	-.491**
F-sozU K-14	-.445**	-.386**	-.293**	-.491**	-.410**	-.420**
SHS	-.638**	-.383**	-.479**	-.656**	-.495**	-.507**

SISE = Singe-Item Self-Esteem Scale; NPI-13 = Narcissistic Personality Inventory 13; BFI-10 = Big Five Inventory 10; SWLS = Satisfaction with Life Scale; RS-11 = Resilience Scale 11; F-sozU K-14 = Questionnaire Social Support; SHS = Subjective Happiness Scale.

**p < .01.

In both groups, life satisfaction, resilience, social support and subjective happiness correlated significantly negatively with depression, anxiety and stress symptoms (see Table 3).

To analyze additional possible differences between the two subsamples regarding the magnitude of described correlations, the effect size Cohen’s q [68] was calculated. Before these calculations, Bonferroni correction (level of significance: p < .05, two-tailed) was conducted to correct multiple comparisons. We found significant group differences for the correlation of resilience with depression, anxiety and stress symptoms (all: q ≈ .200, small effect). They were significantly higher in Facebook users compared to Facebook non-users.

Table 4 presents the relationships between positive variables of mental health and personality traits. In both groups, life satisfaction, resilience, social support and happiness correlated significantly positively with extraversion and self-esteem and significantly negatively with neuroticism. However, there were some differences between the two groups. The following results were only significant in the Facebook user group: positive relationship of life satisfaction, resilience and happiness with narcissism; positive correlation of social support and happiness with agreeableness; positive relationship of happiness and life satisfaction with conscientiousness. Furthermore, resilience and social support correlated significantly positively with openness only in the Facebook non-user group.

**Table 4:** Correlations between personality traits and positive variables of mental health (Facebook users and Facebook non-users).

	Facebook users (N = 790)				Facebook non-users (N = 155)			
	SWLS	RS-11	F-sozU K-14	SHS	SWLS	RS-11	F-sozU K-14	SHS
SISE	.455**	.495**	.348**	.571**	.603**	.586**	.418**	.619**
NPI-13	.153**	.210**	.094	.142**	.149	.126	.075	.027
BFI-10: Extraversion	.308**	.365**	.410**	.384**	.386**	.372**	.414**	.431**
BFI-10: Agreeableness	.064	.050	.106**	.180**	.119	-.051	.038	.148
BFI-10: Conscientiousness	.165**	.351**	.086	.129**	.088	.227**	-.065	.117
BFI-10: Neuroticism	-.290**	-.358**	-.203**	-.413**	-.484**	-.515**	-.279**	-.435**
BFI-10: Openness	-.052	.078	.054	.071	.082	.228**	.242**	.029

SISE = Singe-Item Self-Esteem Scale; NPI-13 = Narcissistic Personality Inventory 13; BFI-10 = Big Five Inventory 10; SWLS = Satisfaction with Life Scale; RS-11 = Resilience Scale 11; F-sozU K-14 = Questionnaire Social Support; SHS = Subjective Happiness Scale.

**p < .01.

### Regressions

#### Personality traits and depression, anxiety, stress symptoms

In the next step, multiple hierarchical regression analyses were employed. First, variables age and gender as well as personality traits narcissism, self-esteem and the “Big Five” traits were taken as independent variables. Variables depression, anxiety and stress, respectively, served as dependent variables. Regression results differed between the two subsamples.

In the Facebook user group, the total model explained 32.8% of the variance of the depression scale, F(9,780) = 42.219, p < .001. Age, gender and openness were not significantly associated with depression symptoms. In contrast, we found significant associations of depression symptoms with self-esteem (standardized beta = -.444), narcissism (standardized beta = .127), extraversion (standardized beta = -.122), and conscientiousness (standardized beta = -.112), all: p < .001. Also, the associations between depression symptoms and neuroticism (standardized beta = .122, p < .01) as well as agreeableness (standardized beta = -.075, p < .05) were significant. Analysis of the confidence intervals showed that self-esteem had the highest association with depression in comparison to the other independent variables of this model. In the Facebook non-user group, depression symptoms were significantly associated with self-esteem (standardized beta = -.366, p < .001) and extraversion (standardized beta = -.202, p < .01). The total model explained 36.8% of the variance of the depression scale, F(9,145) = 9.397, p < .001.

Furthermore, in the Facebook user group, 25.5% of the variance of the anxiety scale was explained by the total model, F(9,780) = 29.666, p < .001. Age (standardized beta = -.066, p < .05), self-esteem (standardized beta = -.273, p < .001), narcissism (standardized beta = .106, p < .01), extraversion (standardized beta = -.105, p < .01) and neuroticism (standardized beta = .270, p < .001) were significantly associated with anxiety symptoms. In the Facebook non-user group, 24.6% of the variance was explained by the total model, F(9,145) = 5.267, p < .001. Significant associations were found between anxiety symptoms and self-esteem (standardized beta = -.264, p < .01) as well as neuroticism (standardized beta = .214, p < .05).

In the next step, the model with the stress scale as the dependent variable was calculated. In the Facebook user group, 32.9% of the variance was explained by the total model, F(9,780) = 42.490, p < .001. Stress symptoms were significantly associated with self-esteem (standardized beta = -.259, p < .001), narcissism (standardized beta = .144, p < .001), agreeableness (standardized beta = -.092, p < .01) and neuroticism (standardized beta = .386, p < .001). In the Facebook non-user group, 31.1% of the variance was explained by the total model, F(9,145) = 7.288, p < .001. Significant associations were found between stress symptoms and self-esteem (standardized beta = -.196, p < .05) as well as neuroticism (standardized beta = .411, p < .001).

#### Positive variables of mental health and depression, anxiety, stress symptoms

Next, multiple regression analyses were calculated with the variables age, gender, life satisfaction, resilience, social support and happiness as independent variables and depression, anxiety and stress symptoms, respectively, as dependent variables.

In the Facebook user group, 46.8% of the variance of the depression scale was significantly explained by the total model, F(6,783) = 114.759, p < .001. Depression symptoms were significantly associated with age (standardized beta = -.084, p < .01), life satisfaction (standardized beta = -.226, p < .001), social support (standardized beta = -.117, p < .001) and happiness (standardized beta = -.397, p < .001). Happiness had the highest association with depression symptoms compared with the other independent variables of the model (no overlaps of the confidence intervals). In the Facebook non-user group, 55% of the variance of the depression scale was explained by the total model, F(6,148) = 30.198, p < .001. Depression symptoms were significantly associated with age (standardized beta = -.187, p < .01), life satisfaction (standardized beta = -.244, p < .05), resilience (standardized beta = -.178, p < .05) and happiness (standardized beta = -.321, p < .001).

Regression results for the anxiety scale showed that in the Facebook user group the total model explained 24.7% of the variance, F(6,783) = 42.890, p < .001. Anxiety symptoms were significantly associated with age (standardized beta = -.140, p < .001), gender (standardized beta = -.115, p < .001), life satisfaction (standardized beta = -.167, p < .001), social support (standardized beta = -.233, p < .001) and happiness (standardized beta = -.116, p < .01). In the Facebook non-user group, 35.9% of the variance was explained by the total model, F(6,148) = 13.787, p < .001. Significant associations were found between anxiety symptoms and age (standardized beta = -.179, p < .05), resilience (standardized beta = -.220, p < .05) as well as happiness (standardized beta = -.221, p < .05).

Furthermore, in the Facebook user group, the total model explained 28.4% of the variance of the stress scale, F(6,783) = 51.860, p < .001. Stress symptoms were significantly associated with age (standardized beta = -.107, p < .01), gender (standardized beta = -.149, p < .001), life satisfaction (standardized beta = -.194, p < .001) and happiness (standardized beta = -.312, p < .001). In the Facebook non-user group, 35.7% of the variance of the stress scale was explained by the total model, F(6,148) = 13.692, p < .001. Significant associations were found between stress symptoms and age (standardized beta = -.152, p < .05) as well as happiness (standardized beta = -.220, p < .05).

## Discussion

The present exploratory study is one of the first investigations to compare Facebook users and Facebook non-users concerning concurrently the relationship between personality traits and mental health (negative and positive variables).

Present results confirm the assumption of systematic differences between Facebook users and Facebook non-users. Facebook users expressed higher values of narcissism (confirmation of Hypothesis 1a) and extraversion than Facebook non-users (confirmation of Hypothesis 1b). These results resemble earlier findings [48,49]. Some studies constituted a positive association between narcissism and Facebook use [24]. They described intensive Facebook users as self-centric, egoistic and self-confident. Simultaneously, these members are sociable and interesting partners for superficial interactions that frequently take place on Facebook [23]. In contrast to earlier results of studies conducted on Facebook [49], in the present study, Facebook users and Facebook non-users did not differ regarding the level of conscientiousness (contradicting Hypothesis 1c). Also no significant differences of agreeableness, neuroticism and openness were found. It should be emphasized that the present study only focused on the use of the SNS Facebook and did not consider other SNSs used by the participants of the present investigation. Thus, all assumptions can only be made for the platform Facebook.

Earlier investigations of the association between the construct self-esteem and the use of SNSs have shown inconsistent findings for several platforms. Mehdizadeh [15] described a negative relationship between Facebook use in the USA and self-esteem. In contrast, Wang et al. [17] found a positive association between interaction on a Chinese SNS and self-esteem. One could argue that these differences could partly be explained by cultural (individualistic culture in the USA, collectivistic culture in China) differences [69]. However, in a study on a German SNS (individualistic culture), Brailovskaia [16] described similar results to Wang et al. [17]. Current results reveal that Facebook users have a higher self-esteem than Facebook non-users. Such inconsistent findings of studies on different SNSs emphasize that in order to understand the relationship between personality and social media use, a more global view—focusing not only on one SNS—is necessary [70]. Therefore, future studies should compare additional countries and their cultural characteristics with respect to the use of different SNSs and self-esteem.

One possible explanation for the described inconsistent findings is the following one. Social needs have been shown to be an important motive for Facebook use [71] and social media use as a whole [70]. Shyness and withholding are characteristics of people with low self-esteem [72]. Such persons rarely initiate social interactions and often feel lonely [73]. The absence of face-to-face confrontations on SNSs makes it easier to interact with other members. People with a low self-esteem profit from the use of SNSs by making new acquaintances and friends and satisfy their need to belong [71,74]. Facebook, for example, presumes a high amount of self-disclosure from its members, which requires a certain level of self-esteem and self-confidence [75]. Facebook members become online-friends and get “Likes” on photos and status updates from each other [76,77]. It can be assumed that the self-esteem of a shy person who does not have many offline-friends and often feels lonely is positively reinforced and increased by getting “Likes” for uploaded photos [46,71]. Probably, participants in studies showing a negative relationship between self-esteem and the use of SNSs use were not long-term users of social platforms. In contrast, studies that postulated a positive association between self-esteem and SNSs use investigated participants with long-term membership. However, this assumption should be examined in longitudinal studies; the development of self-esteem with respect to the duration of membership on different social networks, not only Facebook, could be analyzed over several years.

In the present study, resilience values did not significantly differ between groups. However, Facebook users showed higher values of subjective happiness, life satisfaction and social support than Facebook non-users. One possible explanation is that Facebook users engage in a lot of social interactions [24]. “Likes” and positive comments by online-friends serve as positive feedback satisfying the users’ need for belonging and admiration [23,71]. The feeling of belonging increases their conviction of social support, reinforces their life satisfaction and makes them happier [44,78]. Probably, people who are happy and satisfied with their lives are more willing to upload photos of parties and events and like to share their experiences in status updates more than sad and dissatisfied people. Surely, an interconnection between Facebook use and the level of the positive variables can be assumed. However, such conclusions disregard the fact that both Facebook users and non-users also use other platforms, which could be associated with their mental health variables. Longitudinal research is needed to investigate these assumptions and possible causalities.

Interestingly, the sample of Facebook non-users showed a slightly higher value of depression symptoms than Facebook users (contradicting Hypothesis 2). Earlier studies mostly described a positive relationship between the use of SNSs, e.g., Facebook, and depression [34,79]. However, in some studies no significant relationship was found [33]. The inconsistent results could partly be explained by differing operationalization of Facebook use. A standardized method to measure Facebook use is necessary to shed light on the relationship between depression and Facebook use. Furthermore, in the present study, Facebook users showed higher values of happiness, life satisfaction and social support. This could partly be the reason for their lower depression values compared to Facebook non-users, considering that these variables predict depression [38,80].

However, no significant group differences were found for anxiety and stress symptoms, even though the values of the positive variables—described as significant predictors of anxiety and stress in earlier studies [41,81]–were higher in the Facebook user group. This speaks for the dual-factor model of mental health which describes two interrelated but separate unipolar mental health dimensions (positive and negative) [35–37]. Accordingly, a mentally healthy person has a high level of emotional, psychological and social well-being and a low level of psychopathologies [82].

According to earlier results [40,41], in both groups depression, anxiety and stress symptoms were negatively related to life satisfaction, resilience, social support and happiness (confirmation of Hypothesis 3). People who receive support from their online and/or offline social network develop higher values of resilience and well-being [78,83–85]. Resilient people are more optimistic and happier than those with lower resilience values [40]. They have increased ability to manage stressful and fearful situations that involves individual and social resources [86–88]. Happiness and life satisfaction protect mental health by increasing individual resistance against depression, anxiety and stress [39,42].

Present results demonstrate that depression, anxiety and stress symptoms are associated with certain personality traits. In both groups, depression, anxiety and stress values correlate positively with neuroticism. Neurotic persons are highly sensitive to stress. In stressful, ambiguous situations they tend to show high values of anxiety and depression symptoms and are unable to adequately manage such situations [89]. In contrast, extraversion and self-esteem are negatively associated with depression, anxiety and stress symptoms. Extraverted people have a high need for social interactions. If this need is satisfied, their well-being, happiness and life satisfaction increase [90]. Such persons are perceived as sociable and popular interaction partners who usually receive a lot of social support and are, therefore, well protected against negative mental health.

Only in the Facebook user group, agreeableness correlated significantly negatively with depression and stress symptoms. It can be assumed that Facebook members who behave agreeably towards other users by writing positive comments to status updates also receive more positive feedback to their own online postings [91]. This makes them happier and their feeling of social support increases. This assumption is supported by the positive correlations of agreeableness with happiness and social support found only in the group of Facebook users. Further, only in this group’s regression analyses agreeableness was associated with depression, anxiety and stress symptoms.

In the regression analyses, comparing the Facebook non-user group to Facebook users, fewer traits were associated with the negative mental health variables, especially with anxiety symptoms.

The present study compared Facebook users and Facebook non-users, showing significant differences regarding personality traits and mental health variables. People using Facebook have higher values of, e.g., happiness and life satisfaction as well as personality traits such as narcissism compared to non-users of Facebook. Furthermore, there seems to be a stronger association between personality traits, on the one hand, and depression, anxiety and stress symptoms, on the other hand, in the group of Facebook users. However, does this mean that Facebook use increases positive variables of mental health as well as personality traits? Further studies should investigate this important question raised by the present results. An affirmation of this question would suggest that Facebook use helps to improve mental health making its users more resistant against, e.g., depression. Therefore, it would be beneficial to integrate the use of the SNS Facebook into prevention programs for mental health [79]. Considering the large potential of Facebook in providing social support and satisfying the need to belong [71], the use of this platform could be especially meaningful to people without offline social support. Unlike to face-to-face interaction, in online interactions users can take time to think through their course of action and practice managing stressful situations in order to develop appropriate, resilient behavior.

However, such assumptions would also suggest that traits such as narcissism increase with the use of Facebook. Some authors of earlier studies have already expressed this concern emphasizing that especially younger users of platforms like Facebook show increased narcissism values [92,93]. Longitudinal research will shed more light on these assumptions and suggestions.

### Limitations and further research

The present study had an exploratory character and did not aim to analyze online behavior. It focused only on the membership on the SNS Facebook and not on the use of other SNSs or social media use as a whole.

By distinguishing Facebook users from Facebook non-users, we derived important insights about their diversity. However, some methodical and content-related limitations have to be considered when interpreting the present results.

Participants of the present study were asked about the frequency of SNSs use and about their membership on SNSs as a whole. Results showed that Facebook users were members of up to four other SNSs. Also, not all Facebook non-users disclaimed the use of SNSs. In the Facebook non-use group, the membership on up to three different SNSs was described. 53.5% of the Facebook non-users used SNSs in varying frequency. However, participants were not asked how often they used the individual SNSs. Thus, it cannot be excluded that in both groups time was spent on other SNSs than Facebook, which partly could have caused the found differences.

In order to at least partially constrain this limitation, possible differences between Facebook non-users who answered “never” to use SNSs (*N* = 72) and those who used SNSs in varying frequency (*N* = 83) were investigated by calculating two further MANOVAs. The first analysis included the personality traits as dependent variables. In the second analysis, all mental health variables were included. Both MANOVAs showed no significant results indicating no significant differences within the Facebook non-user group regarding personality traits and mental health variables. Nonetheless, regarding earlier studies which described the reward function of social media use as a whole [70], it is advisable to investigate the use of further SNSs, such as Twitter and Instagram, and not only to focus on the use of only one SNS in future research. This would increase the generalizability of the results.

Considering the significant results of the present study focusing on Facebook use and earlier studies analyzing the association between activities on other SNSs and personality traits [23,94], the investigation of the relationship between behavior on social platforms and mental health seems to be a worthwhile topic of research. To increase the reliability of this investigation, a combination of subjective behavior reports of user habits and objective quantitative measures, e.g., number of online-friends and uploaded images, should be used to analyze online behavior.

Present results reveal differences between Facebook users and people who do not use this SNS. Facebook users have higher values of life satisfaction, social support and subjective happiness than Facebook non-users. Considering that these factors protect mental health, Facebook users should also have a decreased probability to develop mental disorders. However, only a marginally higher value of depression symptoms was found in the Facebook non-user group compared with the Facebook user group. The values of anxiety and stress symptoms did not differ between the groups. Future research should replicate and continue the present investigation, by taking into consideration mental disorders such as obsessive-compulsive disorder or social phobia for comparison. Thereby, more specific measuring instruments than the DASS-21 should be used to measure depression, anxiety and stress.

Furthermore, to prove the generalization of present results, future studies should investigate their replicability using an older sample with a broader age range.

It should also be mentioned that the unequal group size of Facebook users and Facebook non-users limits the drown conclusions. However, the present study investigated only German university students. This partly explains the high amount of Facebook users in the sample, considering that this SNS plays an increased role in young people’s daily life [48]. Also, according to the German Federal Statistical Office in 2015 93% of the German students used SNSs and 7% did not use any SNS [95]. In our sample, 92.4% used SNSs and 7.6% did not use SNSs. Thus, our distribution fits the distribution of the German population.

Additionally, recent studies showed that Facebook gets more and more older users making it more difficult to find a sufficient number of Facebook non-users for comparison studies [96].

Furthermore, as already described, the use of other SNSs and social media use as a whole should be considered in future studies.

## Supporting Information

S1 Dataset Dataset used for analyses in present study. (XLSX)
